# Comparison of 3D endoanal ultrasound and external phased array magnetic resonance imaging in the diagnosis of obstetric anal sphincter injuries

**DOI:** 10.1007/s00330-019-06125-8

**Published:** 2019-03-26

**Authors:** Jaan Kirss, Heikki Huhtinen, Eini Niskanen, Jyrki Ruohonen, Marja Kallio-Packalen, Sarita Victorzon, Mikael Victorzon, Tarja Pinta

**Affiliations:** 1 Division of Digestive Surgery and Urology, Turku University Hospital, Turku, Finland; 2 University of Turku, Turku, Finland; 3 Department of Radiology, Vaasa Central Hospital, Vaasa, Finland; 4 Department of Radiology, Seinäjoki Central Hospital, Seinäjoki, Finland; 5 Department of Surgery, Vaasa Central Hospital, Vaasa, Finland; 6 Department of Surgery, Seinäjoki Central Hospital, Seinäjoki, Finland

**Keywords:** Magnetic resonance imaging, Ultrasonography, Anal canal, Rupture, Postpartum period

## Abstract

**Objectives:**

The gold standard of postpartum anal sphincter imaging has been the 3D endoanal ultrasound (EAUS). Development of magnetic resonance imaging (MRI) has allowed anal sphincter evaluation without the use of endoanal coils. The aim of this study is to compare these two modalities in diagnosing residual sphincter lesions post obstetric anal sphincter injury (OASI).

**Methods:**

Forty women were followed up after primary repair of OASI with both 3D EAUS and external phased array MRI. Details of the anal sphincter injury and sphincter musculature were gathered and analysed.

**Results:**

There was a moderate interrater reliability (*κ* = 0.510) between the two imaging modalities in detecting sphincter lesions, with more lesions detected by MRI. There was a moderate intraclass correlation (ICC) between the circumference of the tear (*κ* = 0.506) and a fair ICC between the external anal sphincter thickness measurements at locations 3 and 9 on the proctologic clock face (*κ* = 0.320) and (*κ* = 0.336).

**Conclusions:**

The results of our study indicate that the use of external phased array MRI is feasible for detecting obstetric anal sphincter lesions postpartum. This allows for imaging of the sphincter defects in centres where EAUS imaging is not available.

**Key Points:**

*• A two centre prospective study that showed external phased array MRI to be a valid imaging modality for diagnosing obstetric anal sphincter injuries.*

**Electronic supplementary material:**

The online version of this article (10.1007/s00330-019-06125-8) contains supplementary material, which is available to authorized users.

## Introduction

The main aetiological factor associated in developing faecal incontinence (FI) is vaginal birth. The aetiology of postpartum FI is multifactorial, with injury to the anal sphincters as the principal cause. Injury to the pudendal nerve, puborectalis muscle, the anal sphincter complex, or the combination of these factors could also play a role in developing postpartum FI [1–3].

Even after a successful initial repair of the sphincter lesion, women with obstetric anal sphincter injury (OASI) have a 50% greater risk of developing faecal incontinence compared to patients without such an injury [4, 5]. In addition to FI, a history of OASI can have a negative effect on sexual function. It has been reported that women with a history of OASI are less likely to plan future pregnancies [6–8].

It is estimated that 1–5% of all vaginal deliveries complicate in perineal tears worldwide [9–11]. There were 53,614 births registered in Finland in 2016; 1.2% of these were complicated by a grade III or IV perineal tear [10].

Vaginal tears are generally repaired by a midwife immediately postpartum. When a sphincter lesion is suspected, the repair is conducted by a gynaecologist or a gastrointestinal (GI) surgeon in operating room conditions with proper anaesthesia [12].

**Fig. 1:**
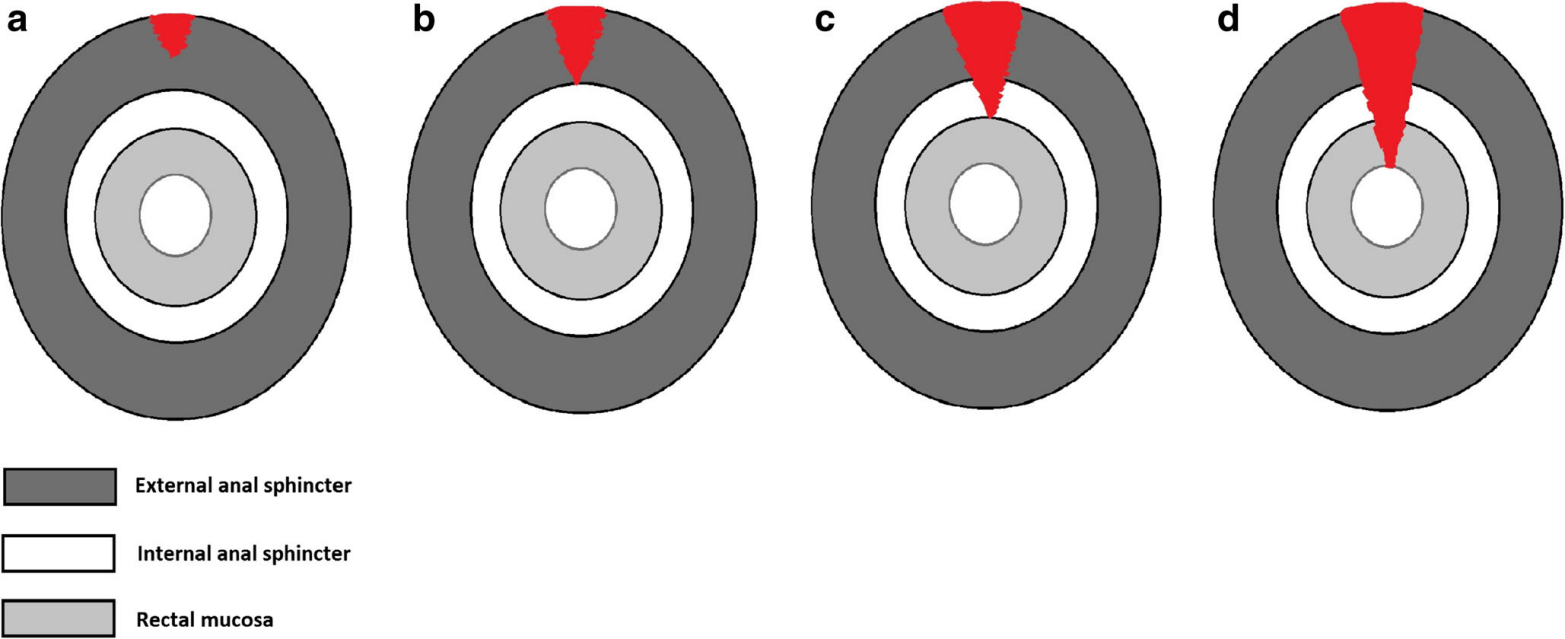
Schematic representation of grade 3–4 perineal tears: (**a**) grade 3a tear, (**b**) grade 3b tear, (**c**) grade 3c tear, (**d**) grade 4 tear

Perineal tears are graded from 1 to 4, according to Sultan et al [13] (Table 1). Grade 3 and 4 perineal tears can also be classified as obstetric anal sphincter injuries (Fig. 1). When a grade 3–4 perineal tear is missed or the initial repair was not successful, the results of secondary repair are usually not encouraging [14–16]. It has been shown that withholding repair until operating room facilities and experienced personnel are available improves the outcomes of primary repair. A delay of up to 24 h has not shown to have a negative effect on the outcomes of primary sphincteroplasty (SP) [17, 18].

**Table 1:** Grading of obstetric injuries according to Sultan et al

Grade 1	Superficial tear of the vaginal mucosa
Grade 2	Tearing of the vaginal mucosa and perineal muscles
Grade 3a	Tear of the EAS, < 50% of the muscle thickness involved
Grade 3b	Tear of the EAS, > 50% of the muscle thickness involved
Grade 3c	Complete EAS and IAS tear
Grade 4	Tear involving the rectal mucosa

Even without an OASI, some women experience a certain degree of FI postpartum, which often subsides after a couple of weeks. This makes the diagnosis of residual OASI challenging without proper imaging.

Endoanal ultrasound (EAUS) has been the gold standard for diagnosing sphincter lesions [19]. Though it is an inexpensive and safe imaging modality, it is operator-specific and not widely available in non-specialist centres. Studies where the results of EAUS and endoanal magnetic resonance imaging (MRI) have been compared have shown that the two modalities are in fact comparable in evaluating sphincter defects [20]. The widespread use of MRI and ever-improving quality of these images has opened up the possibility for imaging the sphincter musculature without the use of endoluminal coils. This has particular clinical value in Finland, where EAUS imaging is available in only 2 of the 16 central hospitals and 3 of the 5 university hospitals, whereas MRI is available in all central and university hospitals.

The aim of this study is to compare 3D EAUS imaging with MRI in the diagnosis of OASI and determine whether external phased array pelvic MRI at 1.5 T is suitable for the diagnosis of OASI.

## Materials and methods

This is a prospective observational study conducted in two Finnish Central hospitals. All women delivering in Vaasa and Seinäjoki Central hospitals between 01/2014 and 08/2017 who had sustained a third or fourth degree OASI diagnosed immediately postpartum were informed of the study. Women suspected of having a missed OASI postpartum were also invited to participate in the study. Written consent was obtained from all participants. All participants were asked to fill out the Wexner incontinence questionnaire. Demographic, obstetric, and follow-up data was collected from electronic patient archives.

All women consenting to participate underwent 1.5 T MR imaging of the rectal musculature 3–12 months after childbirth. Participants were called for a follow-up visit to the surgical outpatient clinic after undergoing MR imaging, 8–12 months postpartum. Upon the follow-up visit, a 3D endoanal ultrasound study was conducted, and patients were informed of the MRI and 3D EAUS study results. All women with symptomatic FI were referred to a physiotherapist for biofeedback and pelvic floor physiotherapy. Women who were still symptomatic 6–8 weeks after starting physical therapy were evaluated for possible future surgical treatment.

The B-K Medical® Ultrasound Scanner Type 2202 with a Type 2050H endoanal probe was used for ultrasound imaging. The imaging was performed by a GI surgeon with the patient in the left lateral position. Later the images were reviewed by two GI surgeons together and analysed for sphincter thickness and scale of sphincter tear, if present.

The MRI devices used were the 1.5 T Discovery MR450, GE Healthcare at Vaasa Central hospital and the Siemens® MAGNETOM® Avanto 1.5 T (software version syngo MR B19) at Seinäjoki Hospital. The MRI procedure was performed with the patient in the resting supine position with the 12-channel body matrix external phased array coil. No intravenous contrast agent, nor rectal or anal contrasting, was used. The details of the MRI procedure are presented in Appendices 1 and 2. The MR images were analysed by two radiologists for the same parameters as the 3D EAUS images. Both of the radiologists were blinded to the 3D EAUS result.

A pilot study was performed that indicated a correlation coefficient (*r*) of 0.788 between the two imaging studies in detecting sphincter lesions. The power analysis revealed that data of 40 women would be needed to prove a correlation (*r* = 0.6) between MRI and 3D EAUS findings with a power of 0.9 (90%) [21].

Patient data was analysed using Microsoft® Excel for MAC version 15.13.1, IBM® SPSS® software Version 23, and SAS System Version 9.4. The intraclass correlation (ICC) values were calculated for continuous variables and interrater reliability (IRR) for categorical variables. The *κ* values from 0.0 to 0.2 indicated a slight agreement, values from 0.21 to 0.40 a fair agreement, values from 0.41 to 0.60 a moderate agreement, values from 0.61 to 0.80 a substantial agreement, and values from 0.81 to 1.0 a perfect agreement [22]. Speakman’s correlation coefficient was used to calculate correlations between ordinal data.

This study was approved by the Hospital District of Southwest Finland Ethics Committee (ETMK 66/1801/2015). The study was registered with Clinicaltrials.gov, identification number NCT 03039374.

## Results

There were 40 women who consented to participate in the study. The study period was from October 2014 to September 2017. The mean age of women was 29.97 years (SD 4.386), with a mean BMI of 24.82 kg/m^2^ (SD 4.729). Most of the women were primiparous (*n* = 25; 62.5%). The median time of birth was at 40 + 2 (range from 37 + 0 to 42 + 6) weeks. Spontaneous births occurred in 52.5% (*n* = 21) cases; in all other cases, the births were vacuum assisted (*n* = 19). Both breech position and shoulder dystocia occurred in one case. Most babies presented in either sinciput (*n* = 16; 40%) or vertex (*n* = 14; 35%) position. The mean time from partum to sphincter repair was 3 h 40 min (SD: 4 h 49 min). The majority of OASIs were repaired by a gynaecologist (*n* = 25; 62.5%); 12 (30.0%) of the tears were repaired by a gastrointestinal surgeon and 3 (7.5%) by a midwife.

Women with an OASI spent an average of 3.35 days (SD: 0.862 days) in hospital postpartum.

The average time from delivery to the MRI study was 235.8 days (SD 138.16 days) and 211.27 days (SD 145.9 days) to the 3D EAUS study (*p* < 0.001).

Of the 40 women analysed, 4 underwent secondary SP performed by a gastrointestinal surgeon. None of the patients were considered for sacral nerve stimulation implantation.

There were 13 EAS tears detected by 3D EAUS and 15 by MRI. The details of the imaging results are presented in Tables 2 and 3. There was a moderate interrater reliability (*κ* = 0.510; *p* < 0.001) between the two imaging modalities when determining types of tears. The inter-class coefficient showed a moderate agreement in determining the circumference of the tear (*κ* = 0.506). There was only a fair ICC between the thickness of the EAS measured at position 3 (*κ* = 0.320) and 9 (*κ* = 0.336) on the proctologic clock face.

**Table 2:** Types of sphincter injuries detected with EAUS and MRI

		*n*	%	Kappa^1^	*p* value^2^
3D EAUS finding	No tear	13	32.5	0.510	< 0.001
	IAS tear	1	2.5		
	EAS tear	13	32.5		
	IAS&EAS tear	13	32.5		
MRI finding	No tear	10	25.0		
	IAS tear	2	5.0		
	EAS tear	15	37.5		
	IAS&EAS tear	13	32.5		

^1^Interrater reliability

^2^Significance of the agreement (McNemar test)

**Table 3:** MRI and 3D EAUS findings of the circumference of the sphincter tear and EAS thickness measurements at positions 9 and 3 on the proctologic clock face

	Mean	Std. deviation	Kappa (ICC)^1^
Mean MRI tear in degrees	63.75	51.375	0.506
Mean 3D EAUS tear in degrees	48.11	38.901	
Mean MRI EAS thickness at 9^2^ (mm)	2.693	0.786	0.336
Mean 3D EAUS EAS thickness at 9 (mm)	2.698	0.794	
Mean MRI EAS thickness at 3^3^ (mm)	2.753	1.078	0.320
Mean 3D EAUS EAS thickness at 3 (mm)	2.838	0.762	

^1^Intraclass coefficient values

^2^Position 9 on the proctologic clock face

^3^Position 3 on the proctologic clock face

The median Wexner score on follow-up was 3 (range 0–11), with a mean score of 4.07 (SD = 3.198).

## Discussion

3D EAUS has been the gold standard for the diagnosis of OASI [23]. 3D EAUS hardware is not widely available in Finland, unlike MR imaging, which is available in all centres that provide obstetric services. There are only five centres in Finland were imaging of the sphincters is possible with 3D EAUS. Women delivering in centres with no 3D EAUS imaging capability were not followed up with imaging modality regardless of having FI symptoms postpartum. Results of this study will enable physicians to conduct imaging of the anal sphincter complex in the absence of EAUS hardware. Studies have shown that mere palpation of the anal sphincters is not accurate enough to diagnose a sphincter lesion [24, 25].

Although there have been studies evaluating the correlation of EAUS with endoanal MR imaging on diagnosing sphincter lesions [20], there have been no studies comparing external phased array MRI and 3D EAUS imaging in diagnosing postpartum OASI. Studies where EAUS imaging is compared to endoanal MRI show that although EAUS is superior in diagnosing IAS defects, endoanal MRI is as sensitive in diagnosing EAS defects [20, 26, 27].

The results of our study indicate that there is a moderate interrater reliability between the MRI and EAUS results in detecting IAS and EAS lesions (Table 2). This is in accordance with previously published research on the comparison of EAUS and endoanal MRI findings [26–28]. In our study, the external phased array MRI was in fact more sensitive, detecting more EAS defects than the EAUS. This is possibly due to the fact that EAUS is unable to differentiate between muscle fibres and scar tissue. In the cases where there was no tear detected on 3D EAUS, the MRI revealed an extensive defect that was replaced by scar tissue. Studies have indicated that extensive scarring in the EAS interferes with the contraction of the EAS, owing to symptoms of FI [29]. It can be argued that the use of MRI could lead to the overdiagnosis of OASI [30]. Even if this was the case, it would not affect the number of patients being treated. It is the view of the authors that SP should be considered for symptomatic patients, no later than 2 years after the initial injury.

**Fig. 2:**
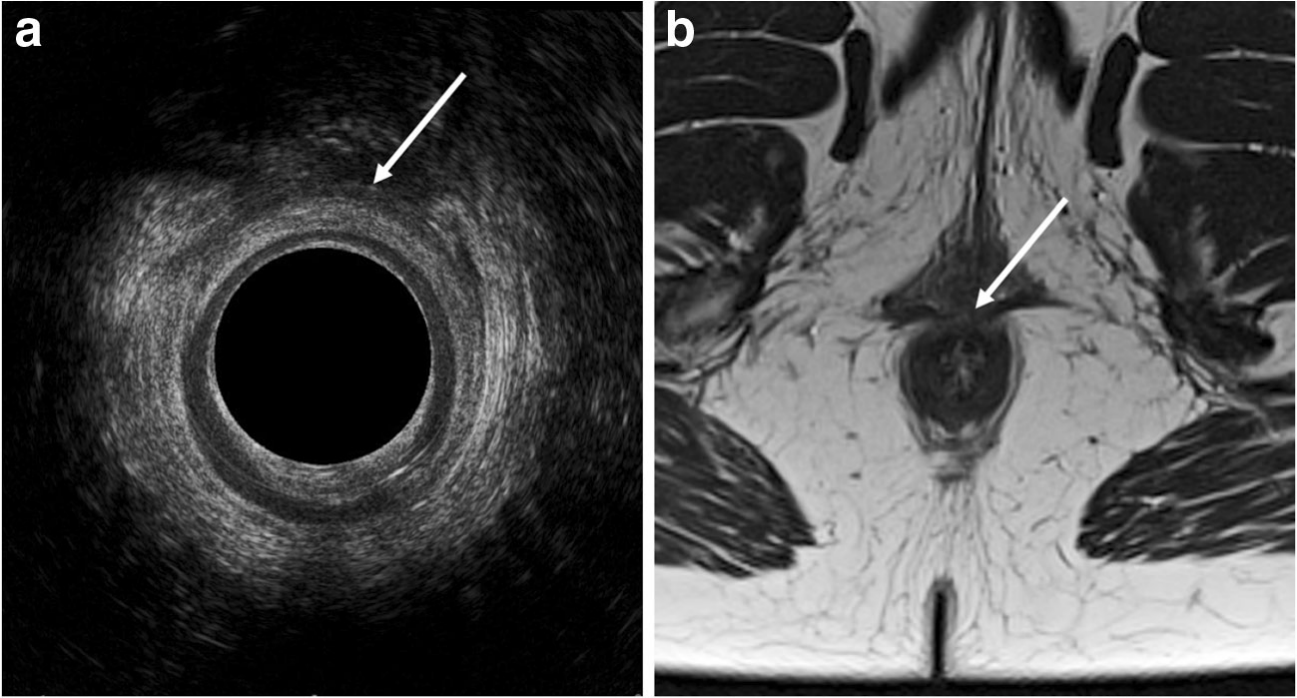
Image of an anterior EAS defect. **a** EAUS image. **b** MR image. The defect is marked with a white arrow

There was a moderate correlation between the two imaging modalities in the measurements of EAS defect circumference. This was expected, since there were no probes, etc. in the anal canal during the MR imaging. This made the evaluation of the defect circumference challenging in the MR images. The fair correlation between EAS sphincter thickness measurements was due to the small size of the structures evaluated. The thickness of the female EAS is 2–3 mm [31, 32]. This makes the measurement of these structures with a 3-mm slice thickness inherently imprecise (Fig. 2). Smaller slice thickness and use of 3D MRI could improve this result. Since the thickness of the EAS has no clinical relevance, this finding should not influence possible treatment strategies.

Young patients with symptomatic OASI should be evaluated for possible sphincter repair before SNS treatment. Results from previously published studies indicate that EAS repair conducted on young patients has a higher level of success [16, 33]. Surgical repair is especially important in patients with a very low perineum or a rectovaginal fistula. Since surgical repair is only available for EAS defects, an external phased array MRI at 1.5 T is sufficient in diagnosing these defects and thus planning surgical treatment for symptomatic patients.

The main limitation of this study was the lack of a control group of healthy women. In addition, the study was performed using only 1.5-T MRI scanners. A 3-T scanner could increase the signal-to-noise-ratio of MR images thus possibly improving the detection of abnormalities. However, since 1.5-T scanners are still more common and more easily available, this study was conducted using only 1.5-T scanners.

Our results indicate that external phased array MRI at 1.5 T is suitable for evaluating the anal sphincter complex postpartum. In a setting where EAUS imaging is not accessible, external phased array MRI can be used to evaluate possible residual sphincter injuries.

## Electronic supplementary material

ESM 1 (DOCX 15 kb)

## Abbreviations

3D EAUS: Three-dimensional endoanal ultrasound
EAUS: Endoanal ultrasound
FI: Faecal incontinence
MRI: Magnetic resonance imaging
OASI: Obstetric anal sphincter injury
