# Supplementary material for: Comparison of 3D endoanal ultrasound and external phased array magnetic resonance imaging in the diagnosis of obstetric anal sphincter injuries
Source: Eur Radiol. 2019 Mar 26;29(10):5717–22. doi: 10.1007/s00330-019-06125-8 (PMC6717181; doi:10.1007/s00330-019-06125-8)
Supplement: Supplementary file 1 — (DOCX 15 kb) [file 330_2019_6125_MOESM1_ESM.docx]

**Appendix 1**

Imaging protocol used in Vaasa Central hospital

T_2_-weighted FRFSE (fast recovery fast spin echo) sequences in sagittal, axial, and coronal planes were imaged with the following parameters: TR/TE 3201/102 (sagittal), 3300/102 (axial), 3034/85 (coronal), ETL 16, FOV 25 x 25 cm, matrix 320 x 320, slice thickness 3 mm with 0.3 mm interslice gap, number of excitations 4, bandwidth 35.41 kHz. Axial slices were obtained perpendicular to the long axis of the anal canal and the coronal slices parallel to it. Total imaging time was 13 minutes.

**Appendix 2**

Imaging protocol used in Seinäjoki Central Hospital

T_2_-weighted turbo spin echo (TSE) sequences in the sagittal, axial, and coronal planes were imaged with the following parameters: TR/TE 4850/108, turbo factor 16, the number of excitations 3, number of slices 32, slice thickness 3mm with a 0.75 mm inter-slice gap (sagittal), TR/TE 4250/99, turbo factor 18, the number of excitations 4, number of slices 25, slice thickness 3mm with a 0.3 mm inter-slice gap (axial), TR/TE 3760/108, turbo factor 20, the number of excitations 5, number of slices 20, slice thickness 3mm with a 0.3 mm inter-slice gap(coronal). The bandwidth 220 Hz/Px, the parallel acquisition techniques GRAPPA with acceleration factor 2, FOV25 x 25 cm and matrix 320 x 320 were used in each of the imaging planes. Axial slices were obtained perpendicular to the long axis of the anal canal and the coronal slices parallel to it. Total imaging time was 15 min 15 s.
